# Fentanyl, Methamphetamine and Polysubstance Use Differentially Affect Locomotor Sensitisation and Social Behaviour in Rats: Psychedelic Treatment Reverses Social Deficits

**DOI:** 10.1111/adb.70132

**Published:** 2026-03-05

**Authors:** Leah M. Salinsky, Kyra C. Diaz, Joshua L. Fox, Shawn M. Panh, Susan M. Ferguson

**Affiliations:** ^1^ Center for the Neurobiology of Addiction, Pain and Emotion University of Washington Seattle USA; ^2^ Addictions, Drug & Alcohol Institute, School of Medicine University of Washington Seattle USA; ^3^ Department of Psychiatry & Behavioral Sciences, School of Medicine University of Washington Seattle USA

**Keywords:** fentanyl, methamphetamine, polysubstance, psychedelic, social interaction

## Abstract

Polysubstance use of opioids and stimulants is increasingly common among individuals with a substance use disorder, yet most researchers examine these substances in isolation. This gap limits our understanding of the effects of polysubstance use and how these differ from single substance use. Here, we examined the impact of single versus polysubstance exposure of fentanyl and methamphetamine on locomotor sensitisation and social behaviour in male and female rats. In addition, as recent evidence has suggested the potential for psychedelic compounds to decrease facets of both opioid and stimulant use disorders, we tested whether the psychedelic R‐(−)2,5‐dimethoxy‐4‐iodoamphetamine (DOI) can reverse drug withdrawal‐induced social deficits. Baseline sociability was assessed in male and female Sprague–Dawley rats using DeepLabCut and Simple Behavioral Analysis (SimBA). Rats then received injections of saline, methamphetamine (1 mg/kg) and/or fentanyl (20 μg/kg) for 14 days, and locomotion was measured. All rats then underwent 10 days of withdrawal followed by a reassessment of sociability. The following day, all subjects received DOI (0.3 mg/kg; 30 min) and were reassessed for sociability. Our results indicate that the development of locomotor sensitisation and drug withdrawal‐induced social deficits vary as a function of drug class, drug history and sex. In addition, acute DOI treatment is sufficient to reverse social deficits as well as enhance social interactions in females. The findings from these experiments suggest a potential therapeutic role of psychedelics in mitigating the social deficits that are associated with withdrawal from polysubstance use of opioids and stimulants.

## Graphical abstract

Polysubstance opioid–stimulant use is increasingly prevalent, yet its behavioral consequences remain poorly understood. We compared single versus combined fentanyl and methamphetamine exposure in male and female rats, assessing locomotor sensitization and sociability during withdrawal. Polysubstance exposure uniquely disrupted social preference, and acute treatment with the psychedelic 5‐HT2A agonist DOI reversed these withdrawal‐induced social deficits, supporting serotonergic psychedelics as potential interventions for social dysfunction in polysubstance use disorder.

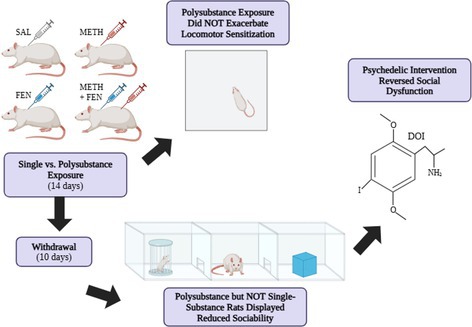

## 1 Introduction

Drug overdose deaths within the United States have drastically risen over the past two decades, climbing by approximately 520% from 1999 to 2023 [1]. During this time period, overdose deaths involving multiple drugs (i.e., polysubstance use) have also increased, especially the combination of opioids and stimulants [2, 3, 4]. Indeed, over 80% of opioid overdose deaths now involve other psychoactive substances, and notably, in 2022, over 50 000 drug overdose deaths were attributed to opioids in combination with stimulants (e.g., methamphetamine). This rise in polydrug use poses a significant public health crisis, as polydrug use amplifies risks of drug–drug interactions and overdose, and adds complexities for prevention and treatment. With drug overdose deaths currently at crisis levels within the United States, polydrug use marks a fourth phase in the overdose epidemic [2, 4, 5]. Despite the high prevalence of polydrug use, most preclinical research has examined opioids or stimulants in isolation, leaving a critical gap in understanding how combined exposure alters behavioural and neurobiological outcomes.

Repeated exposure to certain psychoactive drugs, including stimulants and opioids, can induce locomotor sensitisation, defined as a progressive and enduring increase in the locomotor response to a given drug following repeated administration [6, 7, 8, 9]. Locomotor sensitisation has been heavily used in rodent models to probe neuroadaptations associated with drug use, and some researchers have argued that drug‐induced locomotor sensitisation reflects underlying neuroadaptations relevant to addiction vulnerability, though the extent to which sensitisation directly models addictive behaviour remains debated [10, 11]. Therefore, to address the gap in polysubstance research, we compared the ability of single‐ versus polysubstance treatment of methamphetamine and fentanyl to induce locomotor sensitisation in male and female rats.

Beyond locomotor activity, social behaviours are increasingly recognised as critical modulators of addiction risk. Social isolation exacerbates drug use and relapse vulnerability, whereas positive social interactions serve as protective factors [12, 13]. Conversely, drug use can disrupt social functioning, promoting withdrawal from social networks in a detrimental cycle [13, 14, 15, 16, 17, 18]. Both preclinical and human studies support this bidirectional relationship: Opioid withdrawal reduces preference for social interaction in rodents [16], while chronic opioid use in humans is associated with social dysfunction and isolation [15, 17, 19, 20, 21]. Likewise, repeated stimulant administration impairs social exploration in animal models [18, 22] and has been linked to anhedonia and impaired social cognition in people who use stimulants [23, 24]. Given the rapid increase in fentanyl‐methamphetamine co‐use, it is essential to examine how polysubstance exposure impacts social interaction relative to single‐drug exposure.

Recent research has renewed interest in psychedelic compounds as potential therapeutics for psychiatric disorders, including substance use disorders (SUDs) [25, 26, 27]. Notably, psychedelics have demonstrated efficacy in decreasing facets of both opioid and stimulant use disorders and psychedelic compounds (e.g., lysergic acid [LSD], psilocybin) increase sociability in preclinical models of stress and treatment‐resistant depression [28, 29, 30, 31, 32, 33]. Thus, we evaluated the ability of the psychedelic compound, (−)‐2,5‐dimethoxy‐4‐iodoamphetamine (DOI) to reverse social deficits resulting from withdrawal from chronic drug treatment.

Collectively, these experiments provide the first direct comparison of single‐ versus polysubstance administration of fentanyl and methamphetamine on locomotor sensitisation and social interaction. Further, they explore the therapeutic potential of a psychedelic compound to mitigate drug‐induced social impairments, offering translational insight into novel approaches for addressing polysubstance use disorders.

## 2 Materials and Methods

### 2.1 Animals

Male Sprague–Dawley rats (*n* = 46; Inotiv Inc., Livermore, California, United States) weighing 225–250 g upon arrival and female Sprague–Dawley rats (*n* = 46; Inotiv Inc., Livermore, California, United States) weighing 180–200 g upon arrival were allowed to acclimate for 7 days in a colony room with controlled temperature and humidity on a 12‐h light/dark cycle (lights on 0600–1800 h). Outbred Sprague–Dawley rats were used for these experiments due to their well‐documented response to behavioural tasks and substantial prevalence in substance use disorder research. In addition, Sprague–Dawley rats show high social motivation, making them ideal subjects to investigate drug‐induced social deficits [34]. Rats were pair‐housed according to sex, and weighed and handled daily for at least 5 min by experimenters throughout the study. Food (LabDiet, Irradiated PicoLab) and water were available ad libitum throughout all phases of the studies except during behaviour sessions. Behavioural experiments took place during the light phase of the light/dark cycle and all experiments were carried out in accordance with the *National Research Council Guide for the Care and Use of Laboratory Animals* and with approval from the University of Washington Institutional Animal Care and Use Committee.

### 2.2 Drugs

Fentanyl HCl (National Institute on Drug Abuse, Bethesda, Maryland) administered subcutaneously (*sc*), rac‐Methamphetamine HCl (National Institute on Drug Abuse, Bethesda, Maryland) administered intraperitoneally (*ip*), and (−)‐DOI hydrochloride (Sigma‐Aldrich, CAS‐No. 82864‐02‐6‐, lot 0000336122) administered *sc* were all dissolved in 0.9% NaCl and administered at a volume of 1 mL/kg.

### 2.3 Baseline Sociability Evaluation

Following acclimation to the colony, rats were assessed for social preference over an inanimate novel object using a three‐chamber test; rats were run in the same order across tests. The apparatus for the social interaction test consisted of a plexiglass rectangular, three‐chamber box with dividing walls with an open middle section that allows for free access to each chamber. Each side chamber measured 36 cm × 36 cm × 29 cm and the middle section measured 14 cm × 36 cm × 29 cm. The subject and stimulus rats were placed in the experimental room and allowed to acclimate to room conditions for 15 min prior to experimentation. Prior to testing, the subject rats were allowed to freely explore the three‐chamber box for 5 min. This free exploration period was repeated prior to all subsequent social interaction tests. During testing, a strain, age and sex matched novel stimulus rat was placed under a rectangular perforated plastic crate (24.4 cm × 16.5 cm × 9.7 cm) on one side of the box while a novel object was placed on the other side. Between subjects, the side chambers containing the stimulus rat and the novel object were randomised between the left and right chambers. For each trial, the subject rat was placed in the centre chamber with free access to all three chambers for 10 min. Social preference was scored based on time spent in each chamber. Interaction time with the social stimulus (T_S_) and novel object (T_N_) were quantified, and social preference indexes (I_SP_) were calculated as I_SP_ = (T_S_‐T_N_)/(T_S_ + T_N_) [35, 36].

### 2.4 Tracking Using DeepLabCut

The location of the subject rats within the three‐chamber box during the social interaction test was automatically tracked using the ‘single animal’ class in DeepLabCut (DLC) software version 2.3.10 [37]. A total of 760 training frames were automatically extracted using the k‐means algorithm. A training dataset was then created by manually labeling four points on the rat, including the nose, left ear, right ear and tail base. The labeling was then performed using DLC's ‘napari’ plug‐in, version 0.4.18. The resulting numerical data (CSV format) representing the location of each body part throughout the recorded session for each rat was subsequently entered into SimBA's region of interest (ROI) feature.

### 2.5 Region of Interest Evaluation Using SimBA

The location of the subject rats within the three‐chamber box was evaluated using the Simple Behavioral Analysis (SimBA) toolkit (version 2.8) [38]. Experimental videos and their corresponding tracking data from DLC were imported into the SimBA project and evaluated using the ROI interface where the social chamber, novel object chamber, and centre chamber were defined for each video. ROI data was used to determine how much time the animals spent in the different ROIs.

### 2.6 Locomotor Sensitisation

The psychomotor effects of methamphetamine and fentanyl were measured using an open field activity system (San Diego Instruments, San Diego, California). Following habituation to the colony, rats received a 30 min habituation to the locomotor chambers the day prior to beginning the experimental paradigm. On each test day, rats were moved into an experimental room and allowed to acclimate for 15 min prior to any experimental manipulation. Rats received 14 daily treatments of either methamphetamine + saline, saline + fentanyl, methamphetamine + fentanyl or saline + saline with one injection in the morning (AM, 10:00–11:00 h) and one in the afternoon (PM, 14:00–15:00 h). Injections were spaced 4 h apart so that testing for both drugs occurred during the same phase of the light/dark cycle. Methamphetamine (1 mg/kg, *ip*) was administered in the mornings and fentanyl (20 μg/kg, *sc*) was administered in the afternoons. The 1 mg/kg dose of methamphetamine and 20 μg/kg dose of fentanyl were selected as previous studies have shown increases in drug‐induced locomotion with these doses in Sprague–Dawley rats [6, 7]. Sequential drug administration was utilised in these experiments as this pattern is commonly reported in human use [39, 40]. We chose to utilise a 14‐day drug administration regimen as it provides sufficient time for the drug to induce neuroadaptive and behavioural changes. Several studies with both opioids and stimulants show that around 2 weeks of repeated administration leads to social deficits, negative affect, or anxiety‐like behaviour, which parallels the withdrawal and protracted effects seen clinically [41, 42, 43]. On treatment Days 1, 7 and 14 immediately following treatment, rats were placed into the locomotor chambers for 30 min of locomotor testing. All rats were placed into the locomotor chambers twice a day. The polysubstance group received drug treatment in both time windows. Therefore, to maintain consistency in experimental conditions across groups, the saline control group received two time‐matched locomotor testing sessions per experimental day and the single‐drug groups also received a time‐matched saline session per experimental day.

### 2.7 Assessment of Social Interaction Following Drug Withdrawal

Following the treatment paradigm described above, all subjects were returned to their home cages for 10 days. On day 11, all subjects were reassessed for their social preference with a novel stimulus rat and novel object in the three‐chamber test, as described above.

### 2.8 Evaluating the Ability of DOI to Reduce Social Deficits Resulting From Drug Withdrawal

Twenty‐four hours following the reassessment of social interaction after drug withdrawal, all subjects were treated with the serotonergic psychedelic DOI (0.3 mg/kg, *sc*; 30 min prior) and placed back into the three‐chamber box for an additional test. This dose of DOI was chosen as it has been shown to decrease self‐administration of both stimulants and opioids without impacting other operant behaviours (e.g., latency to first active lever press, inactive lever presses) and does not overtly alter spontaneous locomotion in Sprague Dawley rats [28, 30, 44, 45]. In a separate experiment, a subset of rats (*n* = 6/per sex) that received both methamphetamine and fentanyl were treated with saline (1 mL/kg, *sc*; 30 min prior) instead of DOI.

## 3 Statistical Analysis

### 3.1 Locomotor Activity: Baseline Sex Differences and Effects of Prior Drug Exposure

Analyses were restricted to saline sessions collected from animals in the saline (SAL), single methamphetamine (METH) and single fentanyl (FEN) treatment groups. Single‐drug animals received a saline session temporally matched to the alternate drug exposure session administered to polysubstance‐treated animals. Accordingly, SAL group locomotor data from the afternoon (PM) session was used for comparisons with the single METH‐treated group, whereas SAL group locomotor data from the morning (AM) session was used for comparisons with the single fentanyl‐treated group. For all analyses, saline sessions from drug‐pretreated animals were compared to the corresponding saline sessions from the SAL control group to control for treatment schedule and experimental timing.

To assess baseline sex differences in locomotor activity independent of drug exposure, saline‐treated animals were analysed using two‐way repeated‐measures ANOVA with sex (male, female) as a between‐subjects factor and day (Day 1, Day 7, Day 14) as a within‐subjects factor. Greenhouse–Geisser corrections were applied when violations of sphericity were detected. Main effects of sex and day, as well as sex × day interactions, were evaluated.

To determine whether prior drug exposure altered saline‐induced locomotor activity, separate two‐way repeated‐measures ANOVAs were conducted within each sex comparing saline‐pretreated animals to drug‐pretreated animals. In these analyses, treatment history (SAL vs. METH or SAL vs. FEN) was included as a between‐subjects factor and day as a within‐subjects factor. Main effects of treatment history and day, as well as treatment history × day interactions, were assessed.

Post hoc multiple comparisons were conducted only when a significant interaction involving day was detected, to evaluate time‐dependent effects. When no significant interaction was present, results were interpreted based on omnibus main effects, and no post hoc comparisons were performed.

To characterise within‐session locomotor activity during saline exposure, time‐course analyses were conducted using 3‐min time bins across the full duration of the locomotor sessions. These analyses were performed separately by sex and treatment history to maintain consistency with the experimental design and session timing.

For saline‐treated animals, within‐session time courses were generated for males and females across all three saline exposure days. Because saline animals received both AM and PM saline sessions, these sessions were analysed separately to preserve time matching with drug‐treated groups.

For single‐drug groups, within‐session analyses were restricted to the saline sessions time‐matched to the corresponding drug exposure schedule. Specifically, within‐session time courses were generated for single fentanyl‐treated males and females using their AM saline sessions across all three testing days, and for single methamphetamine‐treated males and females using their PM saline sessions across all three testing days. This approach ensured that saline‐induced locomotor activity was compared under equivalent session timing and handling conditions across treatment groups.

Within‐session locomotor activity was analysed using two‐way repeated‐measures ANOVA with time bin (3‐min bins) and day (Day 1, Day 7, Day 14) as within‐subjects factors. Greenhouse–Geisser corrections were applied when violations of sphericity were detected. Main effects of time bin and day, as well as time bin × day interactions, were evaluated. Post hoc bin‐wise comparisons were conducted only when a significant time × day interaction was detected.

### 3.2 Impact of Daily Drug Treatment on Drug‐Induced Locomotion

Total locomotor activity data was analysed using linear mixed‐effects models to account for repeated measurements within subjects. For both the fentanyl and methamphetamine experiments, sex (male, female) and treatment (saline [SAL], single‐drug [METH or fentanyl], polysubstance [POLY]) were included as between‐subject fixed factors, and day (Day 1, Day 7, Day 14) was included as a within‐subject fixed factor. Animal ID was included as a random intercept to model within‐subject correlations across repeated testing days.

For each dataset, a full‐factorial linear mixed‐effects model including all main effects and interactions (sex × treatment × day) was fit using restricted maximum likelihood (REML) estimation. Fixed effects were evaluated using Wald tests, with statistical significance determined from the associated z statistics and corresponding *p* values derived from the standard normal distribution, as implemented in the *statsmodels* package.

Where significant higher‐order interactions were detected, follow‐up simple‐effects analyses were conducted to decompose these interactions. Specifically, treatment effects were examined at relevant time points and/or within sex, depending on the interaction structure observed, while retaining animal ID as a random intercept. Post hoc comparisons were evaluated using Wald *z* statistics, and *p* values were adjusted for multiple comparisons using the Holm–Bonferroni method. When a factor did not contribute to any significant interaction (e.g., no sex interactions in the fentanyl model), follow‐up analyses were conducted with data collapsed across that factor to maximise power and simplify interpretation.

Within‐session locomotor activity was analysed for the Single‐ and POLY drug‐treated groups in 3‐min time bins using two‐way repeated‐measures ANOVA conducted separately for each sex × treatment group on Day 1, Day 7 and Day 14. Time bin was treated as a within‐subjects factor, with Geisser–Greenhouse correction applied when appropriate. For methamphetamine, within‐session analyses were conducted separately within each sex × treatment group to reflect sex‐dependent effects observed in total locomotion. For fentanyl, within‐session analyses were conducted collapsed across sex (given the absence of sex effects in total locomotion) within each treatment condition. A priori comparisons to Day 1 were evaluated using Dunnett's multiple‐comparisons test when post hoc tests were warranted by significant interaction effects.

### 3.3 Impact of Daily Drug Treatment on Social Interaction

Social behaviour was quantified using a social index, with positive values indicating preference for a novel stimulus rat and negative values indicating preference for a novel object. Social behaviour was assessed at three time points: baseline (BL; prior to any drug or saline exposure), withdrawal (Day 10 following cessation of a 14‐day treatment regimen), and following DOI administration (30 min after DOI pretreatment on Day 11 of withdrawal).

Baseline social index values were analysed using a two‐way analysis of variance (ANOVA) with sex (male, female) and treatment (saline, fentanyl, methamphetamine, polysubstance) as between‐subjects factors. Main effects of sex and treatment, as well as their interaction, were evaluated to confirm equivalence of social behaviour across experimental groups prior to drug exposure.

Following the experimental paradigm, social index data were analysed using linear mixed‐effects models to account for repeated measurements within subjects. Sex (male, female), treatment (saline, fentanyl, methamphetamine, polysubstance) and day (BL, withdrawal, DOI) were included as fixed effects, and animal ID was included as a random intercept. Models were fit using restricted maximum likelihood (REML) estimation. Fixed effects were evaluated using Wald tests, with statistical significance determined from associated z statistics and *p* values. Omnibus effects of treatment were assessed using Wald χ^2^ tests.

Where significant higher‐order interactions involving day were detected, follow‐up simple‐effects analyses were conducted to decompose these interactions. Specifically, treatment groups were compared to saline controls within each time point using estimated marginal means. When no significant interactions involving sex were detected, post hoc analyses were conducted collapsed across sex. To control for multiple comparisons, *p* values from post hoc tests were adjusted using the Holm–Bonferroni method.

### 3.4 Polysubstance‐Only Control Cohort

In a separate cohort, all animals received the polysubstance (POLY) treatment regimen, and social behaviour was assessed at baseline, withdrawal and a saline control test conducted on Day 11 of withdrawal in place of DOI administration. This cohort was used to determine whether withdrawal‐associated social deficits resolved spontaneously in the absence of psychedelic treatment. Social index data from this cohort were analysed using linear mixed‐effects models with day (BL, withdrawal, saline [SAL]) and sex as fixed effects and animal ID as a random intercept. Models were fit using REML estimation, and fixed effects were evaluated using Wald z statistics and corresponding *p* values. Where significant main effects of day were detected in the absence of significant sex × time interactions, comparisons were interpreted relative to baseline collapsed across sex. No sex‐stratified post hoc comparisons were performed when interaction terms did not reach statistical significance.

Baseline social comparisons and within‐session time‐course analyses were performed using GraphPad Prism (version 10.6). Longitudinal analyses of social behaviour and locomotor activity were conducted using linear mixed‐effects models implemented in Python with the statsmodels package. All statistical tests were two‐tailed, and statistical significance was set at α = 0.05.

## 4 Results

### 4.1 Baseline Locomotor Activity and Sex Differences

**FIGURE 1:**
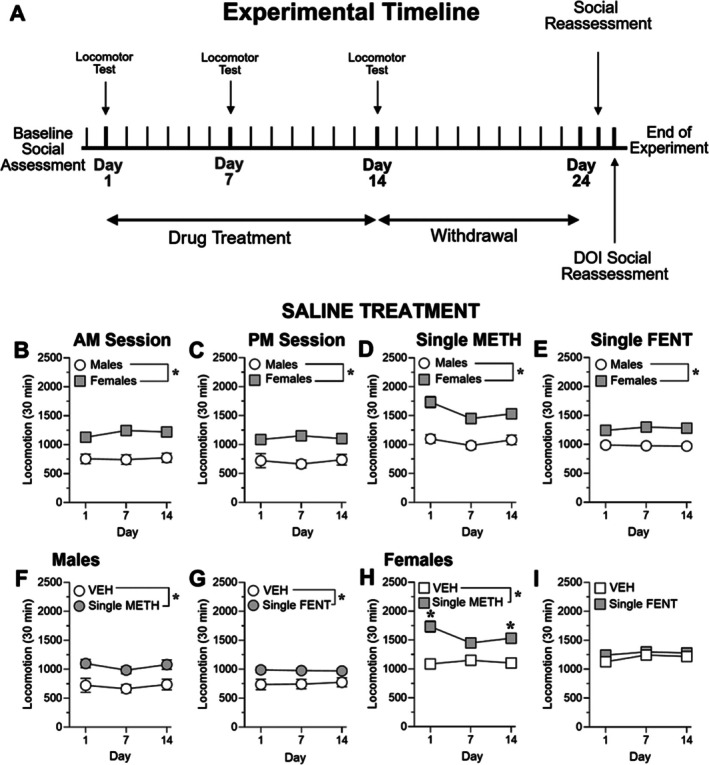
Female rats have higher locomotion than male rats. (A) Experimental timeline. (B,C) Total saline‐induced locomotion of male and female rats in the morning (AM; B) and afternoon (PM; C) treatment sessions. Male rats are symbolised as circles and female rats are symbolised as squares (*p < 0.05 vs. Males, main effect of sex). (D,E) Total locomotion during the saline treatment session for the single METH group (D) and the single FENT group (E). Male rats are symbolised as circles and females are symbolised as squares (*p < 0.05 vs. Males, main effect of sex). (F) Total saline‐induced locomotion of male rats during the VEH session and single METH saline session. (G) Total saline‐induced locomotion of male rats during the VEH session and single FENT saline session. The saline vehicle group is symbolised as white circles and single drug groups are symbolised as shaded circles (*p < 0.05 vs. VEH, main effect of treatment). (H) Total saline‐induced locomotion of female rats during the VEH session and single METH saline session. (I) Total saline‐induced locomotion of female rats during the VEH session and single FENT saline session. The saline vehicle group is symbolised as white squares and single drug groups are symbolised as shaded squares (*p < 0.05 vs. VEH, main effect of treatment, significant interaction of treatment × time). *N* = 10/group.

A timeline of the overall experimental design is shown in Figure 1A. Male and female rats were assigned to one of four treatment groups prior to the start of the experiment: saline (SAL), single methamphetamine (METH), single fentanyl (FEN) or combined methamphetamine + fentanyl (POLY).

### 4.2 Sex Differences in Saline‐Treated Animals

Baseline locomotor activity in saline‐treated animals was examined to assess sex differences independent of drug exposure. Across all treatment cohorts, female rats exhibited significantly higher levels of saline‐induced locomotion compared to males (two‐way repeated‐measures ANOVA; Figure 1B–E). Significant main effects of sex were observed in each cohort (all F_1,18_ ≥ 13.62, *p* < 0.05), while no significant main effects of day or sex × day interactions were detected (all *p* > 0.05). These results indicate a stable sex difference in baseline locomotor activity, with females displaying higher locomotion than males across testing days. Because no sex × day interactions were detected, no post hoc comparisons across days were conducted.

### 4.3 Effects of Prior Drug Exposure on Saline‐Induced Locomotion

To determine whether prior drug exposure altered baseline locomotor activity, saline‐induced locomotion was compared between drug‐pretreated and saline‐pretreated animals within each sex.

In male rats, prior exposure to either methamphetamine or fentanyl significantly increased saline‐induced locomotion relative to saline controls (two‐way repeated‐measures ANOVA; Figure 1F,G). Significant main effects of treatment were observed for both methamphetamine (F_1,18_ = 13.14, *p* < 0.05, Figure 1F) and fentanyl (F_1,18_ = 6.92, *p* < 0.05, Figure 1G), while neither the main effect of day nor the treatment × day interaction reached significance in either comparison (all *p* > 0.05). These findings indicate that prior exposure to either drug elevated baseline locomotor activity in males in a time‐independent manner. Accordingly, no post hoc comparisons across days were conducted.

In female rats, prior methamphetamine exposure significantly increased saline‐induced locomotion compared to saline controls (Figure 1H), as indicated by a significant main effect of treatment (F_1,18_ = 25.30, *p* < 0.05). A significant treatment × day interaction was also detected (F_1.29,23.25_ = 5.26, *p* < 0.05), indicating that the magnitude of this effect varied across testing days. In contrast, prior fentanyl exposure did not alter saline‐induced locomotion in females, as no significant main effects of treatment or treatment × day interactions were observed (Figure 1I; all *p* > 0.05).

Because a significant treatment × day interaction was present only in methamphetamine‐treated females, time‐dependent effects were only examined in this group. No post hoc comparisons were conducted for groups lacking a significant interaction.

To control for potential effects of repeated saline exposure and session timing, within‐session locomotor activity during saline sessions was analysed across Days 1, 7 and 14 using two‐way repeated‐measures ANOVA with time bin (3‐min bins) and day as within‐subjects factors, conducted separately by sex and treatment history. Across all saline conditions, a significant main effect of time bin was consistently observed, reflecting expected within‐session changes in locomotor activity (Supplementary Table S1, Supplementary Figure 1). In contrast, neither the main effect of day nor the time bin × day interaction reached significance in the majority of conditions, indicating stable overall locomotor activity and preserved within‐session patterns across repeated saline exposures.

Notably, no saline condition exhibited a consistent time bin × day interaction across sexes or treatment histories, indicating an absence of sensitisation‐ or habituation‐like changes in within‐session locomotor activity patterns. Accordingly, no post hoc bin‐wise comparisons were performed when interaction effects were not present. These findings confirm that repeated saline exposure and session timing did not systematically alter locomotor activity and therefore did not confound drug‐induced locomotor sensitisation analyses.

### 4.4 Sex‐Dependent Effects of Repeated Methamphetamine Exposure on Locomotor Activity

**FIGURE 2:**
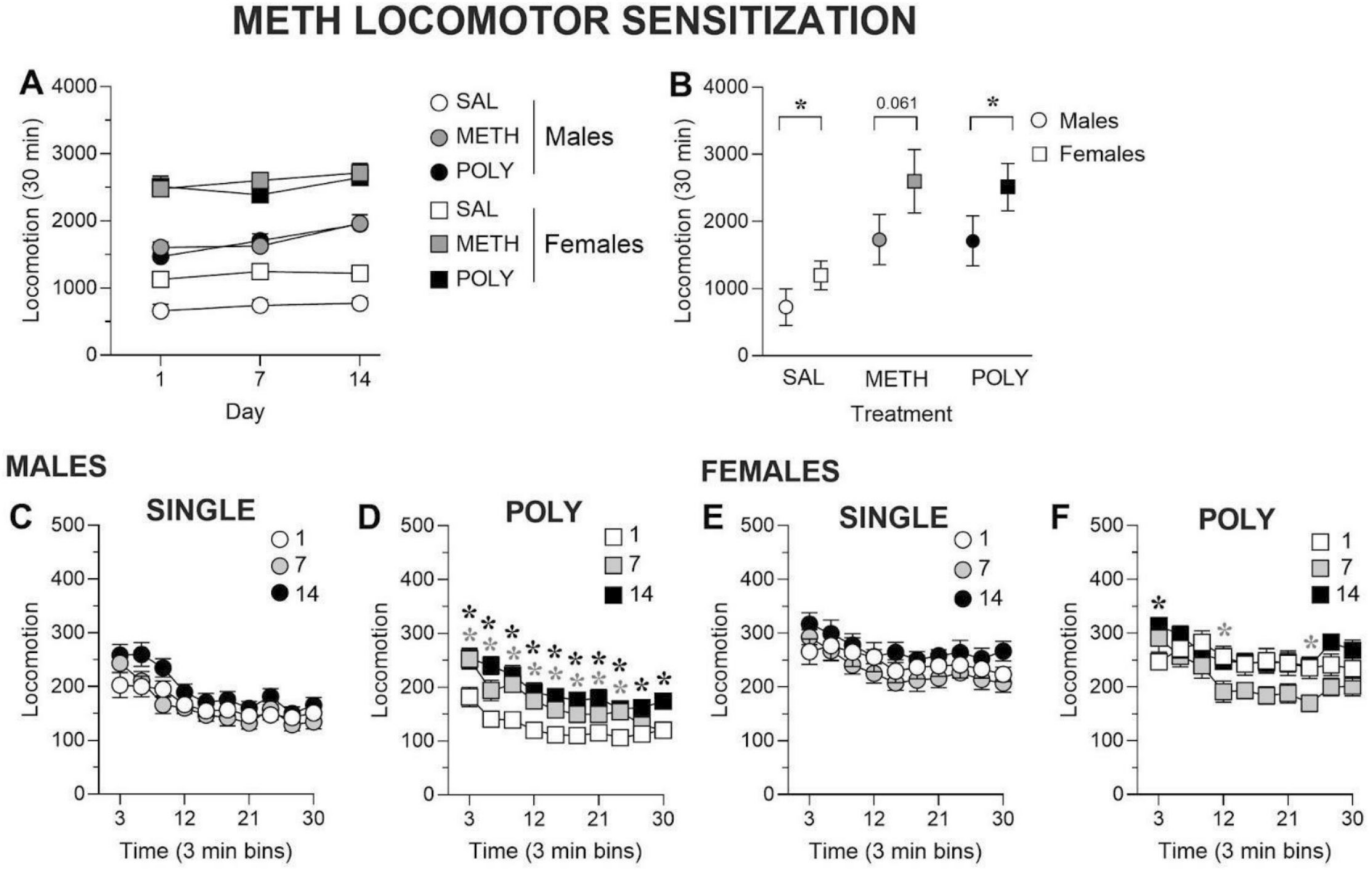
Sex‐dependent effects of repeated methamphetamine exposure on locomotor activity. (A) Total locomotion in male and female rats during repeated METH treatment. Saline control rats are symbolised in white, single METH rats are symbolised in grey and POLY rats are symbolised in black. Males are symbolised as circles and females are symbolised as squares. (B) Total locomotion of male and female rats between treatment groups collapsed across days. Males are symbolised as circles and females are symbolised as squares (*p < 0.05 vs. Males). (C–F) Time course (3‐min bins) of METH‐induced locomotion in males in single (C) and POLY (D) groups and in females in single (E) and POLY (F) groups. Day 1 is symbolised in white, Day 7 is symbolised in grey and Day 14 is symbolised in black. Single‐drug groups are symbolised as circles and POLY groups are symbolised as squares (*(grey) *p* < 0.05, Day 1 vs. Day 7; *(black) *p* < 0.05, Day 1 vs. Day 14, significant interaction of day × time bin). *N* = 10/group.

We assessed the ability of a 14‐day methamphetamine (METH) treatment regimen to induce locomotor sensitisation in single versus polysubstance (POLY) groups by examining locomotor activity on Days 1, 7 and 14. Total locomotor activity following methamphetamine exposure was analysed using a linear mixed‐effects model including sex, treatment and day as fixed factors and animal ID as a random intercept. The full‐factorial model revealed a significant sex × treatment interaction (*z* = −2.65, *p* = 0.008, Figure 2A), indicating that the magnitude of methamphetamine‐induced locomotor activity differed between males and females. No significant main effect of day and no interactions involving day (treatment × day or sex × treatment × day) were detected (all *p* > 0.1, Figure 2A), indicating an absence of time‐dependent changes in locomotor activity across repeated testing sessions.

Because no effects involving day were observed, the sex × treatment interaction reflects differences in the overall magnitude of methamphetamine‐induced locomotion rather than differences in sensitisation across days. Accordingly, treatment effects were interpreted collapsed across day (i.e., averaging each animal's responses across Days 1, 7 and 14). Under these conditions, both METH and POLY groups exhibited significantly greater locomotor activity than saline controls, and overall locomotor activity differed between male and females (Figure 2A), consistent with a sex‐dependent difference in the expression of methamphetamine‐induced hyperlocomotion.

To interpret sex effects within each treatment condition, the mixed‐effects model was parameterised with saline (SAL) and males as the reference levels. In this framework, the main effect of sex reflects the male–female difference specifically within the SAL group, whereas sex × treatment interaction terms indicate whether this sex difference is modified by drug treatment.

A significant main effect of sex was detected (*z* = −3.07, *p* = 0.002, Figure 2B), indicating that within the saline condition, males exhibited significantly lower locomotor activity than females, establishing a baseline sex difference in locomotor output. In the methamphetamine (METH) group, the sex × treatment interaction did not reach statistical significance (*p* = 0.061, Figure 2B), indicating that the sex difference in locomotor activity was not reliably altered by METH exposure relative to saline, although the direction of the effect was consistent with lower activity in males.

In contrast, a significant sex × treatment interaction was observed for the polysubstance (POLY) group (*z* = −2.65, *p* = 0.008, Figure 2B), demonstrating that the sex difference in locomotor activity was significantly altered under POLY conditions relative to SAL. Specifically, males exhibited markedly lower locomotor activity than females following polysubstance exposure (Figure 2B), indicating an enhanced sex difference in the expression of drug‐induced locomotion.

Together, these findings show that a baseline sex difference in locomotor activity is present under saline conditions, persists under methamphetamine exposure and is significantly amplified following polysubstance treatment. Importantly, these effects reflect differences in absolute locomotor activity levels between males and females within each treatment group rather than differences in sensitisation across testing days, as no interactions involving day were detected in the mixed‐effects analysis.

Thus, while methamphetamine produced robust hyperlocomotion relative to saline and this effect differed by sex, it did not induce time‐dependent locomotor sensitisation under the experimental conditions tested. Polysubstance exposure did not further potentiate methamphetamine‐induced locomotor activity, and sex‐dependent differences were maintained across days. To further characterise within‐session patterns of locomotor activity following repeated methamphetamine exposure, locomotion was analysed in 3‐min time bins using two‐way repeated‐measures ANOVA with time bin (within‐session bin) and day (Day 1, Day 7, Day 14) as factors, conducted separately within each sex and treatment group.

### 4.5 Within‐Session Locomotor Analyses: Males

In the single METH group, a significant main effect of time bin was observed (Geisser–Greenhouse corrected; F_3.41,30.70_ = 37.51, *p* < 0.0001, Figure 2C), while the main effect of day was not significant (F_1.88,16.92_ = 2.198, *p* > 0.05, Figure 2C). No significant interaction between time bin × day was found (F_4.53,40.78_ = 1.692, *p* > 0.05, Figure 2C). Accordingly, no post hoc bin‐wise comparisons were performed, indicating that the within‐session pattern of methamphetamine‐induced locomotor activity remained stable across repeated testing days in males receiving METH alone.

In the POLY group, a significant main effect of time bin was also observed (F_3.099,27.89_ = 26.64, *p* < 0.0001, Figure 2D), along with a significant main effect of day (F_1.793,16.14_ = 19.24, *p* < 0.0001, Figure 2D) and a significant time bin × day interaction (F_5.638,50.75_ = 2.673, *p* = 0.027, Figure 2D) indicating that the temporal distribution of locomotor activity across the session differed across testing days. Post hoc analyses revealed significantly greater locomotor activity on Day 7 compared to Day 1 during all time bins except the final bin (27–30 min), and significantly greater activity on Day 14 compared to Day 1 across all time bins (*p* < 0.05; Figure 2D).

Together, these analyses indicate that repeated methamphetamine exposure alone did not alter the within‐session organisation of locomotor activity in male rats, whereas polysubstance exposure was associated with time‐dependent changes in within‐session locomotor patterns across testing days. However, because within‐session analyses were conducted separately within each treatment group, no direct statistical comparisons between METH and POLY groups were performed. Moreover, consistent with the absence of treatment × time bin interactions in the mixed‐effects analysis of total locomotor activity, these findings suggest that polysubstance exposure modifies the temporal organisation of methamphetamine‐induced locomotion rather than producing robust increases in overall activity across days.

### 4.6 Within‐Session Locomotor Analyses: Females

In female rats treated with METH alone, a significant main effect of time bin was observed (Geisser–Greenhouse corrected; F_3.742,33.68_ = 6.763, *p* < 0.05, Figure 2E), while neither the main effect of day (F_1.295,11.66_ = 1.807, *p* > 0.05, Figure 2E) nor the time bin × day interaction (F_5.612,50.51_ = 0.7515, *p* > 0.05, Figure 2E) reached statistical significance. These findings indicate that repeated methamphetamine exposure did not alter the within‐session organisation of locomotor activity across testing days in females receiving METH alone.

In the POLY group, a significant main effect of time bin was also observed (F_3.389,30.50_ = 7.987, *p* = 0.003, Figure 2F) whereas the main effect of day did not reach statistical significance (F_1.253,11.28_ = 3.853, *p* = 0.0681, Figure 2F). However, a significant time bin × day interaction was detected (F_4.846,43.61_ = 2.707, *p* = 0.034, Figure 2F), indicating modest day‐dependent changes in the temporal distribution of locomotor activity across the session. Post hoc analyses revealed significantly decreased methamphetamine‐induced locomotion on Day 7 relative to Day 1 in time bins 12 and 24, as well as significantly increased locomotor activity during the first time bin on Day 14 compared to Day 1 (Dunnett‐adjusted; *p* < 0.05; Figure 2F).

Together, these findings indicate that repeated methamphetamine exposure does not produce robust within‐session reorganisation of locomotor activity in female rats treated with METH alone. While polysubstance exposure was associated with statistically significant changes in within‐session activity patterns, these effects were limited in magnitude, bidirectional in direction and not accompanied by consistent increases in total locomotor activity across days. Accordingly, these results suggest subtle modulation of within‐session locomotor dynamics rather than sensitisation‐like behavioural changes in females.

### 4.7 Repeated Fentanyl Exposure Induces Locomotor Sensitisation Independent of Sex

**FIGURE 3:**
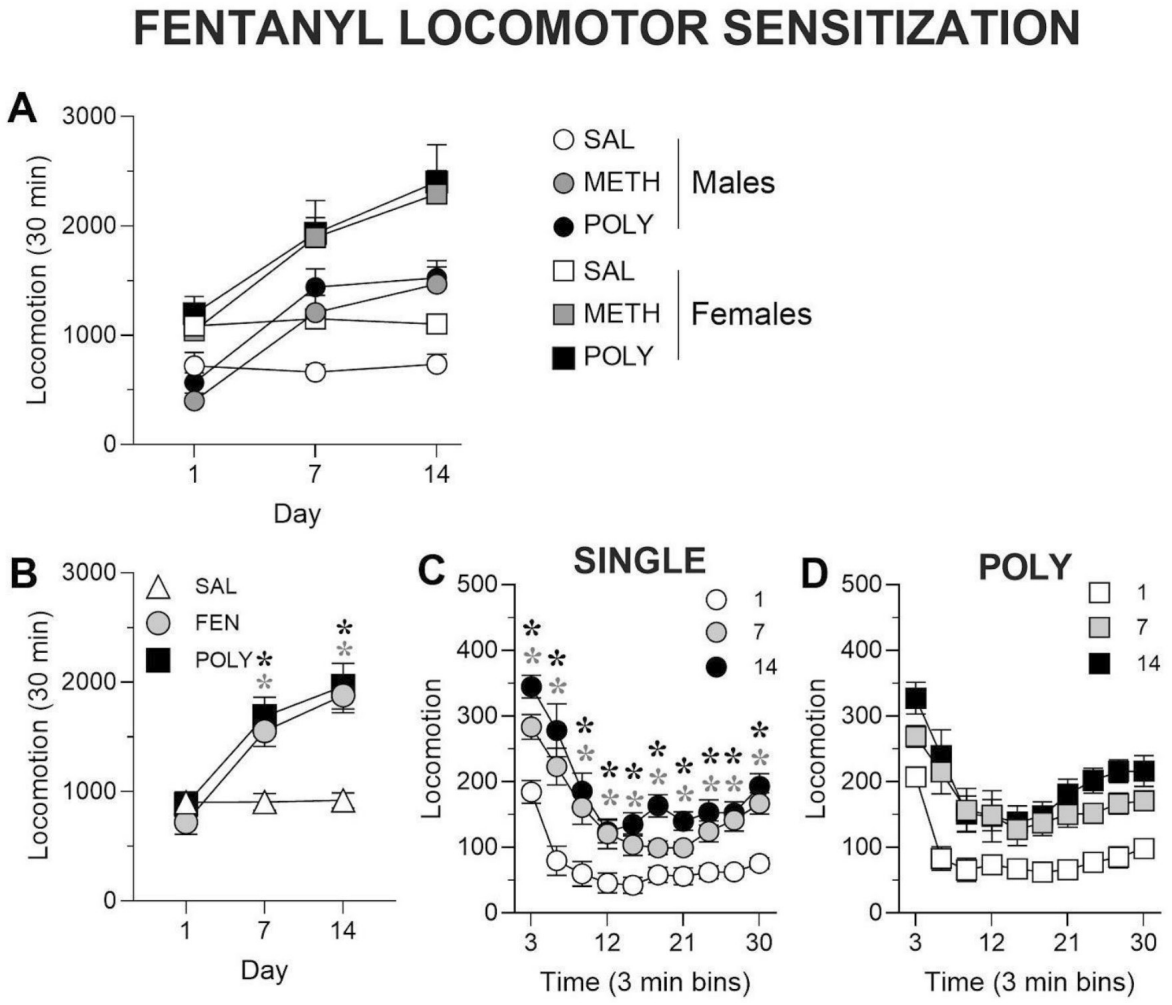
Repeated fentanyl exposure induces locomotor sensitisation independent of sex. (A) Total locomotion in male and female rats during repeated fentanyl treatment. Saline control rats are symbolised in white, single fentanyl rats are symbolised in grey and POLY rats are symbolised in black. Males are symbolised as circles and females are symbolised as squares. (B) Total locomotion during repeated fentanyl treatment collapsed by sex. Saline control rats are symbolised in white triangles, single fentanyl rats are symbolised as grey circles, and POLY rats are black squares. (*(grey) *p* < 0.05, single fentanyl vs. saline; *(black) *p* < 0.05, POLY vs. saline, significant interaction of day × treatment). (C,D) Time course of FENT‐induced locomotion in single (C) and POLY (D) groups collapsed by sex. Day 1 is symbolised in white, Day 7 is symbolised in grey and Day 14 is symbolised in black. Single groups are symbolised as circles and POLY groups are symbolised as squares. (*(grey) *p* < 0.05, Day 1 vs. Day 7; *(black) *p* < 0.05, Day 1 vs. Day 14, significant interaction of day × time bin). *N* = 20/group, 10/sex.

We also examined the ability of a 14‐day fentanyl regimen to produce locomotor sensitisation in single‐drug and polysubstance (POLY) groups. Total locomotor activity following fentanyl exposure was analysed using a linear mixed‐effects model including sex, treatment and day as fixed factors and animal ID as a random intercept. The full‐factorial model revealed a significant treatment × day interaction (*z* = −5.74, *p* < 0.001, Figure 3A), indicating that treatment effects on locomotor activity differed across testing days. Males exhibited a nonsignificant trend toward lower locomotor activity compared to females (*z* = −1.73, *p* = 0.084, Figure 3A), and no higher‐order interactions involving sex reached statistical significance. Because no significant interactions with sex were detected, post hoc comparisons were performed across all animals without stratification by sex.

On Day 1, no significant differences in locomotor activity were observed between treatment groups (FEN vs. SAL: *z* = −0.25, *p* = 0.802; POLY vs. SAL: *z* = 0.28, *p* = 0.781, Figure 3B), indicating comparable baseline responses to the first drug exposure. In saline‐treated animals, locomotor activity remained stable across testing days (Day 7 vs. Day 1: *z* = 0.03, *p* = 0.98; Day 14 vs. Day 1: *z* = 0.15, *p* = 0.88, Figure 3B), confirming the absence of time‐dependent changes in control animals. By Day 7, both FEN‐ and POLY‐treated animals exhibited significantly elevated locomotor activity relative to SAL controls (FEN vs. SAL: *z* = 5.08, *p* < 0.001; POLY vs. SAL: *z* = 4.91, *p* < 0.001, Holm‐adjusted, Figure 3B), while no significant differences were observed between the FEN and POLY groups (*z* = 0.56, *p* = 0.579, Figure 3B). A similar pattern was observed on Day 14, with FEN‐ and POLY‐treated animals again showing significantly greater locomotor activity compared to SAL controls (FEN vs. SAL: *z* = 5.45, *p* < 0.001; POLY vs. SAL: *z* = 4.91, *p* < 0.001, Holm‐adjusted, Figure 3B), and no differences detected between FEN and POLY conditions (*z* = 0.44, *p* = 0.657, Figure 3B).

Planned within‐group contrasts were conducted to directly assess sensitisation as an increase in activity relative to each group's initial response. In the fentanyl‐only group, locomotor activity was significantly increased on both Day 7 (*z* = 7.41, *p* < 0.001) and Day 14 (*z* = 10.33, *p* < 0.001) compared to Day 1. Similarly, in the polysubstance group, activity was elevated on Day 7 (*z* = 7.17, *p* < 0.001) and Day 14 (*z* = 9.62, *p* < 0.001) relative to Day 1. In contrast, saline‐treated animals showed no change across days (Day 7 vs. Day 1: *z* = 0.03, *p* = 0.98; Day 14 vs. Day 1: *z* = 0.15, *p* = 0.88). These results indicate that fentanyl‐induced hyperlocomotion emerges over repeated exposure and is not further potentiated by polysubstance treatment.

Although males exhibited a nonsignificant trend toward lower overall locomotor activity compared to females (*z* = −1.73, *p* = 0.084, Figure 3A), the absence of significant sex × treatment or sex × day interactions indicates that sensitisation developed comparably across sexes. Thus, fentanyl‐induced locomotor sensitisation is largely sex‐independent under the present experimental conditions and is not exacerbated by prior methamphetamine exposure.

To further characterise within‐session patterns of locomotor activity following repeated fentanyl exposure, locomotion was analysed in 3‐min time bins using two‐way repeated‐measures ANOVA with time bin (within‐session bin) and day (Day 1, Day 7, Day 14) as factors, conducted separately within each sex and treatment group.

### 4.8 Within‐Session Locomotor Analyses: Fentanyl (Collapsed Across Sex)

Because sex did not significantly interact with treatment or day in the mixed‐effects analysis of total fentanyl‐induced locomotor activity, within‐session analyses were conducted with data collapsed across sex.

In animals treated with fentanyl alone, significant main effects of time bin (Geisser–Greenhouse corrected; F_3.337,63.40_ = 46.72, *p* < 0.0001, Figure 3C) and day (F_1.940, 36.85_ = 45.77, *p* < 0.0001, Figure 3C) were observed, indicating expected within‐session variation in locomotor activity and an overall increase in activity magnitude across testing days (Figure 3B). A time bin × day interaction was also detected (F_6.692,127.2_ = 2.667, *p* = 0.0144, Figure 3C), indicating that the distribution of activity across the session differed modestly across days. Post hoc analyses showed that locomotor activity was significantly elevated on both Day 7 and Day 14 relative to Day 1 across all time bins (Dunnett‐adjusted; *p* < 0.05, Figure 3C), reflecting a general increase in fentanyl‐induced activity throughout the session with repeated exposure.

In animals receiving polysubstance treatment, significant main effects of time bin (F_3.910,74.30_ = 23.04, *p* < 0.0001, Figure 3D) and day (F_1.728,32.83_ = 29.80, *p* < 0.0001, Figure 3D) were likewise observed, indicating within‐session variation and increased overall locomotor activity across days. However, the time bin × day interaction was not significant (F_6.538,124.2_ = 1.433, *p* = 0.2022, Figure 3D), suggesting that the relative pattern of activity across the session remained stable across testing days despite increases in activity magnitude. Accordingly, no bin‐wise post hoc comparisons were performed for the polysubstance group.

Together, these findings indicate that repeated fentanyl exposure increases overall locomotor activity across testing days, consistent with the development of locomotor sensitisation. While a time bin × day interaction was detected in the fentanyl‐only condition, this effect was modest and accompanied by broad increases in activity across the session rather than selective or localised changes. In the polysubstance condition, sensitisation was expressed primarily as an increase in activity magnitude without detectable changes in within‐session organisation. Because within‐session analyses were conducted separately within each treatment group, no direct statistical comparisons between fentanyl‐only and polysubstance groups were made. Accordingly, these results are best interpreted as describing differences in how increased locomotor activity is distributed within sessions rather than evidence of qualitatively different or enhanced sensitisation in either condition.

### 4.9 Withdrawal‐Associated Decreases in Sociability Following Polysubstance Exposure Are Reversed by DOI

Social preference was quantified using a social index, with positive values indicating preference for a novel stimulus rat and negative values indicating preference for a novel object. Social behaviour was assessed at three time points (factor of day): baseline (BL; prior to any drug or saline exposure), during withdrawal (Day 10 following cessation of a 14‐day treatment regimen), and following DOI administration (30 min after DOI pretreatment on Day 11 of withdrawal).

**FIGURE 4:**
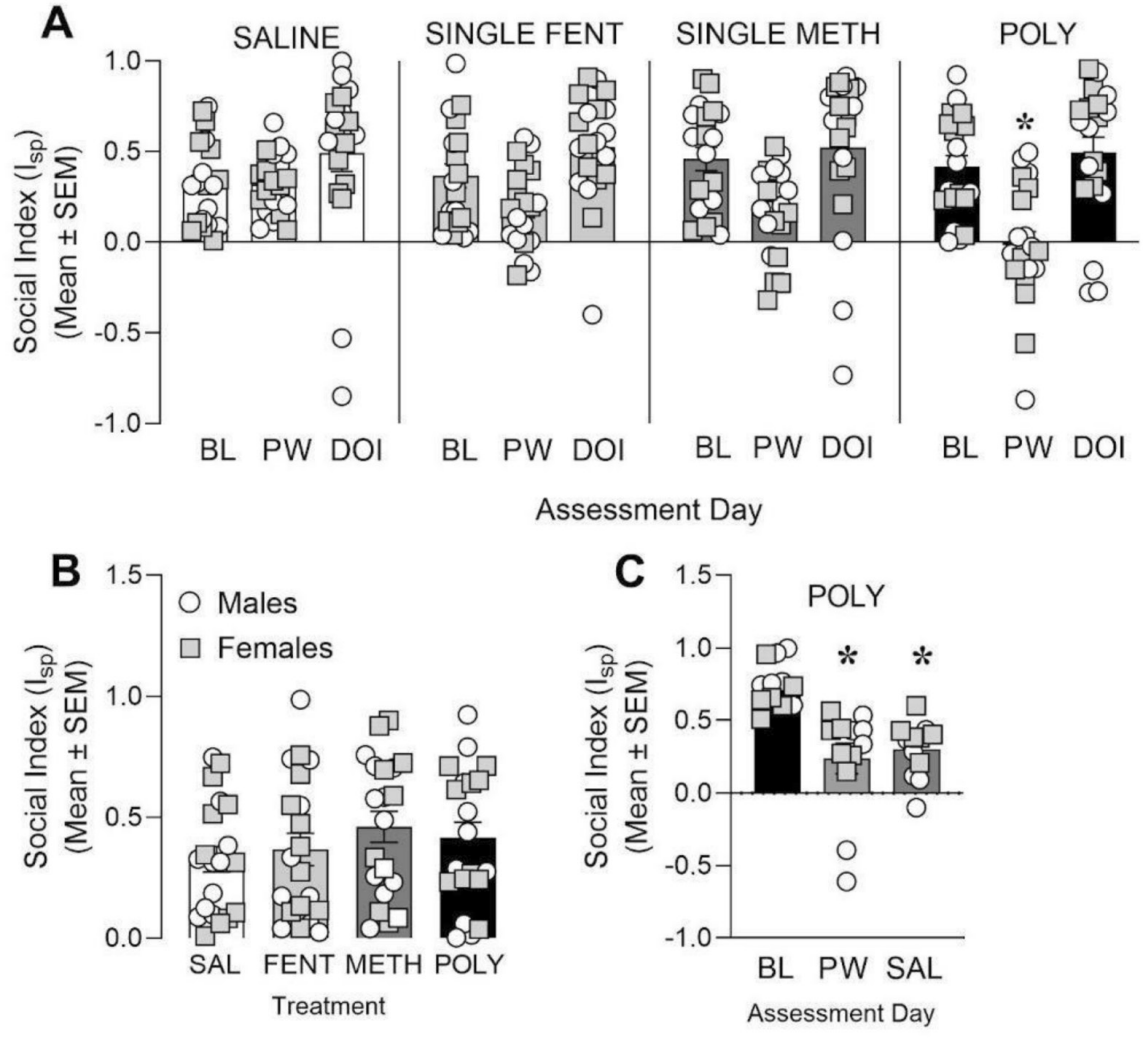
Withdrawal from polysubstance treatment reduces sociability in male and female rats in a manner that is reversed by psychedelic treatment. (A) Social indices of male and female rats within each respective treatment group at baseline (BL), after 10 days of withdrawal (PW), and following acute pretreatment with DOI. (*p < 0.05 vs. saline PW, significant interaction of time × treatment), *N* = 20/group, 10/sex. Male rats are symbolised as circles and female rats are symbolised as squares. (B) Social indices of all rats at baseline (i.e., prior to any treatment). Male rats are symbolised as circles and female rats are symbolised as squares. (C) Social indices of a male and female rats that received POLY treatment at baseline (BL), after 10 days of withdrawal (PW), and following acute treatment with saline (SAL). (*p < 0.05 vs. BL, main effect of time). *N* = 12/group, 6/sex. Male rats are symbolised as circles and female rats are symbolised as squares.

To confirm baseline equivalence across experimental groups prior to treatment, baseline social index values were analysed using a two‐way ANOVA with sex and treatment Group (SAL, FEN, METH, POLY) as between‐subjects factors. There was no significant sex × treatment interaction (F_3,72_ = 0.056, *p* = 0.983, Figure 4B), indicating that baseline social preference did not differ as a function of treatment group differently across sexes. There was also no significant main effect of sex (F_1,72_ = 0.032, *p* = 0.860, Figure 4B), indicating comparable baseline social preference between males and females. Likewise, no significant main effect of treatment was detected (F_3,72_ = 0.839, *p* = 0.477, Figure 4B), demonstrating that baseline social index values did not differ among the SAL, FEN, METH or POLY groups.

The mean baseline social index was 0.387 for males and 0.398 for females, with a mean difference of −0.011 (95% CI −0.138 to 0.115, Figure 4B), further confirming negligible sex differences at baseline. Together, these results indicate that social behaviour was equivalent across sexes and treatment groups prior to any pharmacological manipulation.

Social index data were subsequently analysed using a linear mixed‐effects model including sex, treatment and day as fixed factors and animal ID as a random intercept to account for repeated measurements within subjects. The full factorial model revealed a significant main effect of day (*z* = 6.50, *p* < 0.001, Figure 4A), indicating that social preference varied across testing sessions. No significant main effect of treatment was detected (Wald χ^2^ = 1.33, *p* = 0.72, Figure 4A), indicating no overall group difference in social index when averaged across time points. Additionally, there was no main effect of sex (*z* = 0.22, *p* = 0.82, Figure 4A), indicating no apparent sex difference in social index.

Importantly, a significant day × treatment interaction was observed (*z* = 1.84, *p* = 0.038, Figure 4A), indicating that changes in social preference across days differed as a function of treatment. No higher‐order interactions involving sex reached statistical significance (all *p* > 0.1, Figure 4A), indicating that treatment‐related changes in social behaviour were comparable between males and females. Accordingly, post hoc analyses were conducted collapsed across sex.

To investigate the significant day × treatment interaction, drug‐treated groups were compared to SAL controls within each time point using Holm‐adjusted *p* values. At baseline, social index values did not differ across treatment groups (all *z* < 1, Holm‐adjusted *p* > 0.05, Figure 4A). During withdrawal, POLY‐treated animals exhibited a significant reduction in social index relative to SAL controls (*z* = −2.37, Holm‐adjusted *p* = 0.018, Figure 4A), whereas FEN and METH groups did not differ from SAL (all Holm‐adjusted *p* > 0.05, Figure 4A). Following DOI administration, social index values in POLY‐treated animals no longer differed from SAL controls (z = −0.30, *p* = 0.77, Figure 4A), and no treatment‐related group differences were detected at this time point (all Holm‐adjusted *p* > 0.05, Figure 4A). Together, these findings indicate that polysubstance exposure selectively disrupts social preference during withdrawal and that acute DOI administration is associated with normalisation of social preference relative to saline controls.

To determine whether POLY‐induced social deficits resolve spontaneously or could be attributed to repeated testing, a separate cohort of male and female rats received the POLY treatment regimen and was assessed at baseline, during withdrawal, and following saline administration in place of DOI. Social index data were analysed using a linear mixed‐effects model with day and sex as fixed effects and animal ID as a random intercept.

The model revealed a significant main effect of day (*p* < 0.001, Figure 4C), indicating that social preference differed across testing sessions. In contrast, there was no significant main effect of sex (*z* = −0.86, *p* = 0.392, Figure 4C) and no significant sex × day interaction (all *p* > 0.05, Figure 4C), demonstrating that changes in social behaviour over time were comparable between males and females. Accordingly, follow‐up comparisons across day were conducted collapsed across sex.

Post hoc analyses showed a robust decrease in social index values during withdrawal relative to baseline (*z* = −5.10, *p* < 0.001; Figure 4C), indicating the emergence of a pronounced social deficit following POLY exposure. Social indices remained significantly reduced following saline administration compared to baseline (*z* = −4.52, *p* < 0.001; Figure 4C), with no evidence of recovery.

Importantly, the stability of reduced social index values across repeated testing sessions indicates that POLY‐induced social deficits are not attributable to habituation or repeated task exposure, but instead reflect a persistent impairment that does not resolve spontaneously in the absence of pharmacological intervention.

## 5 Discussion

In this set of experiments, we aimed to evaluate how single versus polysubstance exposure to methamphetamine and/or fentanyl impacts locomotor sensitisation and social behaviour in male and female Sprague–Dawley rats. Overall, our findings demonstrate that the behavioural consequences of repeated drug exposure depend on drug class, sex and drug history, and that alterations in locomotor sensitisation and social behaviour are dissociable outcomes. Notably, polysubstance exposure did not exacerbate locomotor sensitisation but uniquely disrupted social behaviour during withdrawal.

### 5.1 Locomotor Sensitisation Depends on Drug Class and Sex but Is Not Exacerbated by Polysubstance Exposure

Repeated methamphetamine exposure produced sex‐dependent effects on locomotor behaviour. Across treatment conditions, females exhibited higher absolute locomotor activity during methamphetamine testing sessions than males. Because baseline activity also differed between sexes, this finding should be interpreted as a difference in overall activity levels rather than evidence of a greater drug‐induced effect size in females. In contrast, males showed greater sensitivity to repeated drug exposure. Notably, females did not exhibit time‐dependent increases in methamphetamine‐induced locomotion, consistent with prior work demonstrating sex‐dependent differences in stimulant responsivity [46, 47, 48]. Together, these findings indicate that sex is a primary determinant of methamphetamine‐induced locomotor responses.

Across both single‐drug and polysubstance conditions, repeated methamphetamine did not produce robust locomotor sensitisation as defined by progressive, uniform increases in total locomotor activity across testing days. Although within‐session analyses revealed statistically significant effects in some conditions, these effects were modest in magnitude, inconsistent in direction, and not accompanied by corresponding increases in total activity in mixed‐effects models. In male rats, polysubstance exposure is associated with alterations in within‐session activity patterns across testing days whereas males receiving methamphetamine alone did not exhibit significant reorganisation of within‐session locomotor activity. In females, within‐session effects were minimal following methamphetamine alone and limited following polysubstance exposure. Importantly, these analyses were conducted within treatment groups and were not designed to directly compare temporal activity patterns between methamphetamine‐only and polysubstance conditions. Accordingly, within‐session findings should be interpreted as descriptive refinements of locomotor expression rather than evidence of enhanced or accelerated sensitisation. Together, these findings indicate that polysubstance exposure does not exacerbate methamphetamine‐induced locomotor sensitisation under these experimental conditions. Rather, polysubstance history may subtly influence how locomotor activity is distributed within sessions without amplifying overall behavioural output.

In contrast to methamphetamine, repeated fentanyl exposure produced robust increases in locomotor activity across testing days in both single‐drug and polysubstance treated animals. Mixed‐effects analysis revealed a significant treatment × day interaction, indicating that fentanyl‐induced locomotor sensitisation emerged over repeated exposure rather than being present at the initial testing session. Importantly, the magnitude of fentanyl‐induced locomotion did not differ between fentanyl‐only and polysubstance‐treated animals, demonstrating that sequential administration with methamphetamine did not potentiate fentanyl sensitisation.

Within‐session analyses were consistent with this interpretation. When data were collapsed across sex, significant main effects of time bin and day were observed in both fentanyl‐only and polysubstance groups, reflecting expected within‐session variation in activity and overall increases in locomotor output across days. A modest time bin × day interaction was detected in the fentanyl‐only condition, indicating some redistribution of activity across the session with repeated exposure; however, this effect was accompanied by broad increases in locomotor activity across all time bins rather than selective or localised changes. In contrast, the polysubstance group did not exhibit a significant time bin × day interaction, suggesting that increases in fentanyl‐induced activity were expressed primarily as changes in overall magnitude, with the temporal pattern of activity within the session remaining stable.

Together, these findings indicate that fentanyl‐induced locomotor sensitisation is predominantly characterised by increases in activity magnitude across repeated exposures. While modest changes in within‐session distribution were observed in the fentanyl‐only group, these effects were limited and should not be overinterpreted as evidence of major reorganisation of locomotor behaviour. Polysubstance exposure did not amplify either the magnitude or the temporal expression of fentanyl‐induced sensitisation. Thus, fentanyl and methamphetamine produce distinct locomotor adaptation profiles, and although polysubstance history can subtly shape the expression of drug‐induced activity, it does not consistently exacerbate canonical locomotor sensitisation.

To our knowledge, these are the first experiments to investigate the effect of sequential single‐ versus polysubstance administration on locomotor sensitisation in both male and female rats. These results appear to contrast with previous studies reporting additive effects of opioids and stimulants on locomotor activity [49, 50]. Several methodological factors may underlie these discrepancies. Prior work has largely examined simultaneous co‐administration of stimulants with heroin or morphine, whereas our design employed sequential exposure and focused on fentanyl, a high‐potency synthetic opioid with distinct pharmacokinetic and receptor‐binding properties [49, 50, 51, 52]. Sequential versus concurrent drug administration likely engages dopaminergic and opioid receptor systems differently, contributing to divergent behavioural outcomes. Additionally, strain and species differences may also be relevant, as much of the existing literature was conducted in mice or in other strains of rats (e.g., Long Evans), which display different activity levels at baseline and may have differing drug sensitivities compared to Sprague–Dawley rats [53]. As most previous preclinical studies were conducted exclusively in males, the inclusion of both sexes revealed sex‐dependent effects that may have been obscured in male‐only cohorts. Finally, we timed methamphetamine administration following the end of the dark/active cycle and fentanyl prior to the light/inactive cycle to approximate clinically relevant usage patterns as stimulants sustain wakefulness while opioids tend to promote sedation [39, 40]. However, circadian timing of drug exposure has been shown to impact drug reward and may play a role in the differences presented in our findings and previous reports [54]. By implementing these factors, our findings extend current literature by modelling polysubstance use in a translationally relevant framework. However, future work is needed to systematically evaluate how drug class, sequence and circadian timing interact with sex to modify drug‐induced behaviours.

### 5.2 Polysubstance Exposure Uniquely Disrupts Social Behaviour During Withdrawal

In contrast to locomotor outcomes, social behaviour was selectively disrupted by polysubstance exposure. Withdrawal from polysubstance administration produced a robust reduction in social preference that was observed in both male and female rats, whereas withdrawal following methamphetamine or fentanyl exposure alone did not significantly alter social behaviour relative to saline controls. These results indicate that combined stimulant–opioid exposure uniquely compromises social functioning and that this vulnerability is not sex dependent under the present experimental conditions.

The dissociation between locomotor and social outcomes is notable, as drug classes and sex exerted strong effects on locomotor behaviour but not on social preference. This pattern suggests that neural systems underlying social behaviour may be differentially sensitive to polysubstance exposure compared to those governing locomotor activation. Preclinically, the combination of opioids and stimulants often produces additive behaviours despite distinct mechanisms of action [49, 50]. However, there is substantial convergence of mu opioid receptor and dopamine signalling within the nucleus accumbens, a key regulator of both drug reward and social behaviour. Positive social interactions and investigation of novel social partners elicit dopamine release within this region [55], and prior work has demonstrated dopaminergic dysfunction following polysubstance, but not single‐substance, self‐administration of fentanyl and methamphetamine in male rats [56]. Thus, we hypothesise that polysubstance exposure may impair dopamine signalling within mesolimbic circuits, reducing the salience of social stimuli during withdrawal. Future studies will be necessary to directly test how polysubstance exposure alters dopamine dynamics in response to social interaction.

### 5.3 DOI Reverses Polysubstance‐Induced Social Deficits Without Enhancing Sociability

Acute pretreatment with the serotonergic psychedelic DOI normalised social preference in polysubstance‐treated animals, restoring behaviour to levels comparable to saline controls. Importantly, DOI did not increase social preference above baseline levels in animals that did not exhibit withdrawal‐induced deficits, indicating that its effects were specific to reversing existing impairments rather than enhancing sociability. This interpretation is further supported by a control cohort in which saline administration during withdrawal failed to restore social preference, demonstrating that recovery was likely not attributable to repeated testing or spontaneous resolution of deficits.

Previous studies have demonstrated the efficacy of psychedelic serotonin (5‐HT) 2A receptor (5‐HT_2A_R) agonists to promote positive sociability under stressful conditions, but psychedelic compounds have yet to be evaluated for drug‐induced social withdrawal [31, 32]. Hence, our results with DOI present the first preclinical evidence demonstrating the ability of a psychedelic compound to reverse social deficits resulting from drug withdrawal in both male and female rats in this experimental paradigm.

DOI is thought to exert its primary function through agonism of the 5‐HT_2A_R. 5‐HT_2A_R activation may impact attentional processing, and DOI at higher doses than that utilised in this study can alter visual and temporal processing [57, 58]. Therefore, while this dose of DOI does not impact operant responding in other models or alter spontaneous movements, future research is necessary to examine the effects of DOI on sensory processes within the context of the three‐chamber test [30, 44, 45]. A few limitations of the present study should be noted. We employed a single dose of DOI, which does not allow for the evaluation of dose–response relationships and narrows the scope of interpretation. An additional limitation includes the within‐subjects testing design. Although saline controls argue against habituation or spontaneous recovery, future studies employing multiple doses of DOI, additional vehicle controls and counterbalanced testing designs will be important to further define the specificity and durability of DOI's effects.

Lastly, while DOI was employed in these studies as an investigational psychedelic due to an extensive preclinical profile, DOI has yet to be formally studied in humans [59]. However, the psychedelic compound psilocybin is currently under clinical investigation for OUD (NCT06796062, NCT06810310) and methamphetamine use disorder (NCT06899594, NCT05322954). Preclinical investigations of serotonergic psychedelics in translational models of polysubstance use will provide valuable insights for future clinical interventions. As psychedelics continue toward clinical development, additional research is needed to establish the therapeutic efficacy of psychedelics to mitigate the many facets of polysubstance use and evaluate the neurobiological and molecular mechanisms through which these compounds act.

## 6 Conclusion

This study provides one of the first comprehensive evaluations of sequential single‐ versus polysubstance exposure to methamphetamine and fentanyl in both male and female rats. Our findings demonstrate that polysubstance exposure does not exacerbate locomotor sensitisation but uniquely disrupts social behaviour during withdrawal, and that acute psychedelic treatment can selectively reverse these social deficits. Together, these data highlight the dissociable behavioural consequences of polysubstance use and support further investigation of serotonergic psychedelics as potential interventions for social dysfunction associated with substance use disorders.

## Author Contributions

L.M.S., K.C.D. and J.L.F. performed behavioural studies. S.M.P provided technical and intellectual inputs. L.M.S. and S.M.F. conducted data analysis, designed the project and wrote the manuscript. All authors approved the manuscript.

## Funding

This work was supported by National Institute on Drug Abuse R01DA58930, P30DA048736, T32DA007278, R25DA057786.

## Conflicts of Interest

The authors declare no conflicts of interest.

## Supporting information


**Table S1:** Across saline sessions, locomotor activity exhibited a robust main effect of time in all groups (all *p* < 0.0001), reflecting expected within‐session locomotor dynamics. In contrast, no consistent main effects of day or time × day interactions were detected, indicating stable locomotor patterns across repeated saline exposures. A single exception was observed in single methamphetamine–treated males, where a modest time × day interaction was detected, suggesting subtle variation in within‐session organisation across days. Importantly, no group exhibited changes consistent with locomotor sensitisation during saline sessions, supporting the conclusion that sensitisation effects observed in drug‐treated conditions reflect pharmacological effects rather than repeated testing or circadian confounds.
**Figure S1:** Time courses of saline‐induced locomotion in male (A–D) and female (E–H) rats across saline vehicle treatment for the saline and single‐drug treatment groups. (A,E) Time course of saline‐induced locomotion of male and female rats in the morning (AM) treatment session. (B,F) Time course of saline‐induced locomotion of male and female rats in the single FENT group during saline treatment. (C,G) Time course of saline‐induced locomotion of male and female rats in the afternoon (PM) treatment session. (D,H) Time course of saline‐induced locomotion of male and female rats in the single METH group during saline treatment. Male rats are symbolised as circles and female rats are symbolised as squares. Day 1 is symbolised in white, Day 7 is symbolised in grey and Day 14 is symbolised in black (*(grey) p < 0.05, Day 1 vs. Day 7; *(black) p < 0.05, Day 1 vs. Day 14). N = 10/group.

## Data Availability

The data that support the findings of this study are available from the corresponding author upon reasonable request.
