# Supplementary material for: Fentanyl, Methamphetamine and Polysubstance Use Differentially Affect Locomotor Sensitisation and Social Behaviour in Rats: Psychedelic Treatment Reverses Social Deficits
Source: Addict Biol. 2026 Mar 5;31(3):e70132. doi: 10.1111/adb.70132 (PMC12963791; doi:10.1111/adb.70132)
Supplement: Supplementary file 1 — Table S1: Across saline sessions, locomotor activity exhibited a robust main effect of time in all groups (all p < 0.0001), reflecting expected within‐session locomotor dynamics. In contrast, no consistent main effects of day or time × day interactions were detected, indicating stable locomotor patterns across repeated saline exposures. A single exception was observed in single methamphetamine–treated males, where a modest time × day interaction was detected, suggesting subtle variation in within‐session organisation across days. Importantly, no group exhibited changes consistent with locomotor sensitisation during saline sessions, supporting the conclusion that sensitisation effects observed in drug‐treated conditions reflect pharmacological effects rather than repeated testing or circadian confounds. Figure S1: Time courses of saline‐induced locomotion in male (A–D) and female (E–H) rats across saline vehicle treatment for the saline and single‐drug treatment groups. (A,E) Time course of saline‐induced locomotion of male and female rats in the morning (AM) treatment session. (B,F) Time course of saline‐induced locomotion of male and female rats in the single FENT group during saline treatment. (C,G) Time course of saline‐induced locomotion of male and female rats in the afternoon (PM) treatment session. (D,H) Time course of saline‐induced locomotion of male and female rats in the single METH group during saline treatment. Male rats are symbolised as circles and female rats are symbolised as squares. Day 1 is symbolised in white, Day 7 is symbolised in grey and Day 14 is symbolised in black (*(grey) p < 0.05, Day 1 vs. Day 7; *(black) p < 0.05, Day 1 vs. Day 14). N = 10/group. [file ADB-31-e70132-s001.docx]

| **Group** | **Session** | **Sex** | **Main effect of Time** | **Main effect of Day** | **Time × Day interaction** | **Interpretation** |
| --- | --- | --- | --- | --- | --- | --- |
| Saline | AM | Female | F(4.08,36.69)=51.25, p<0.0001 | F(1.69,15.22)=2.42, p=0.128 | F(5.72,51.43)=2.29, p=0.052 | Stable across days |
| Saline | PM | Female | F(4.44,39.97)=45.69, p<0.0001 | F(1.46,13.17)=1.29, p=0.295 | F(5.04,45.33)=0.83, p=0.535 | Stable across days |
| Saline | AM | Male | F(3.83,34.46)=95.79, p<0.0001 | F(1.23,11.08)=1.00, p=0.358 | F(4.96,44.69)=1.30, p=0.282 | Stable across days |
| Saline | PM | Male | F(3.30,29.66)=66.00, p<0.0001 | F(1.55,13.96)=1.87, p=0.195 | F(4.16,37.48)=1.84, p=0.139 | Stable across days |
| Single METH | Saline | Female | F(3.88,34.96)=45.55, p<0.0001 | F(1.47,13.25)=1.66, p=0.226 | F(5.66,50.90)=1.36, p=0.250 | Stable across days |
| Single METH | Saline | Male | F(4.16,37.44)=94.85, p<0.0001 | F(1.41,12.65)=1.74, p=0.215 | F(5.40,48.61)=3.07, p=0.015 | Modest pattern shift |
| Single Fentanyl | Saline | Female | F(5.09,45.84)=109.2, p<0.0001 | F(1.16,10.46)=4.31, p=0.059 | F(4.76,42.81)=0.80, p=0.550 | Stable across days |
| Single Fentanyl | Saline | Male | F(4.59,41.32)=87.72, p<0.0001 | F(1.26,11.33)=4.48, p=0.051 | F(4.90,44.11)=2.27, p=0.065 | Stable across days |

Supplementary Table 1: Across saline sessions, locomotor activity exhibited a robust main effect of Time in all groups (all p < 0.0001), reflecting expected within-session locomotor dynamics. In contrast, no consistent main effects of Day or Time × Day interactions were detected, indicating stable locomotor patterns across repeated saline exposures. A single exception was observed in single methamphetamine–treated males, where a modest Time × Day interaction was detected, suggesting subtle variation in within-session organization across days. Importantly, no group exhibited changes consistent with locomotor sensitization during saline sessions, supporting the conclusion that sensitization effects observed in drug-treated conditions reflect pharmacological effects rather than repeated testing or circadian confounds.


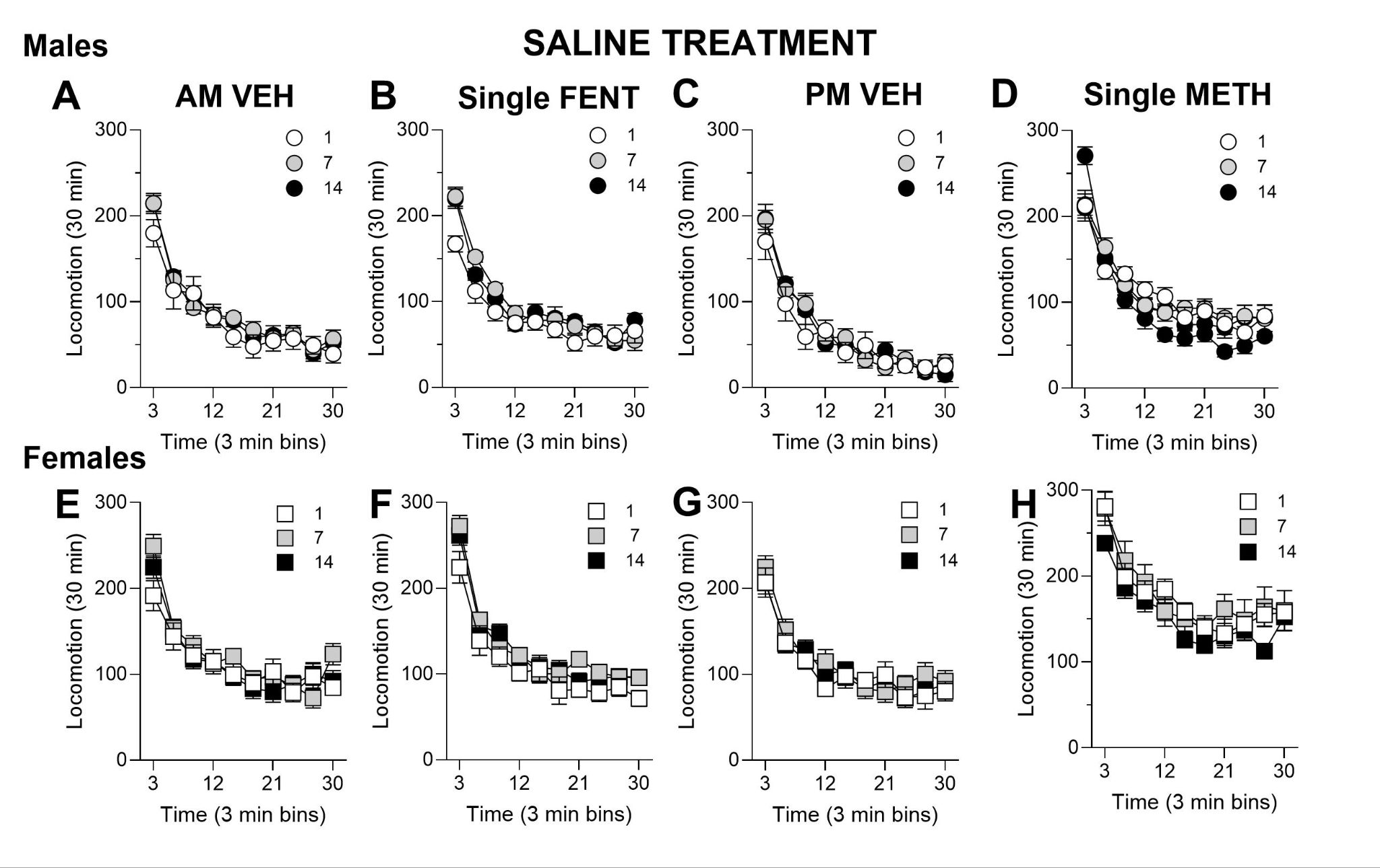


Supplementary Figure 1: Time courses of saline-induced locomotion in male (A-D) and female (E-H) rats across saline vehicle treatment for the saline and single-drug treatment groups. (A,E) Time course of saline-induced locomotion of male and female rats in the morning (AM) treatment session. (B,F) Time course of saline-induced locomotion of male and female rats in the Single FENT group during saline treatment . (C,G) Time course of saline-induced locomotion of male and female rats in the afternoon (PM) treatment session. (D,H) Time course of saline-induced locomotion of male and female rats in the Single METH group during saline treatment. Male rats are symbolized as circles and female rats are symbolized as squares. Day 1 is symbolized in white, Day 7 is symbolized in grey and Day 14 is symbolized in black (*(grey) p < 0.05, Day 1 vs. Day 7; *(black) p < 0.05, Day 1 vs. Day 14). N =10/group.
